# Using Metal-Organic Framework HKUST-1 for the Preparation of High-Conductive Hybrid Membranes Based on Multiblock Copolymers for Fuel Cells

**DOI:** 10.3390/polym15020323

**Published:** 2023-01-08

**Authors:** Ivan Gorban, Nieves Ureña, María Teresa Pérez-Prior, Alejandro Várez, Belén Levenfeld, Carmen del Río, Mikhail Soldatov

**Affiliations:** 1 The Smart Materials Research Institute, Southern Federal University, Sladkova Street 178/24, 344090 Rostov-on-Don, Russia; 2 Departamento de Ciencia e Ingeniería de Materiales e Ingeniería Química, IAAB, Universidad Carlos III de Madrid, Avda. de la Universidad, 30, Leganés, 28911 Madrid, Spain; 3 Instituto de Ciencia y Tecnología de Polímeros (ICTP-CSIC), C/Juan de la Cierva, 3, 28006 Madrid, Spain

**Keywords:** polysulfone, multiblock copolymer, metal-organic frameworks, impedance spectroscopy, fuel cells

## Abstract

Novel proton-conducting hybrid membranes consisting of sulfonated multiblock copolymer of polysulfone and polyphenylsulfone (SPES) reinforced with a HKUST-1 metal-organic framework (MOF) (5, 10, and 20 wt. %) were prepared and characterized for fuel cell applications. The presence of the MOF in the copolymer was confirmed by means of FE-SEM and EDS. The hybrid membranes show a lower contact angle value than the pure SPES, in agreement with the water uptake (WU%), i.e., by adding 5 wt. % of the MOF, this parameter increases by 20% and 40% at 30 °C and 60 °C, respectively. Additionally, the presence of the MOF increases the ion exchange capacity (IEC) from 1.62 to 1.93 m_equiv_H^+^ g^−1^. Thermogravimetric analysis reveals that the hybrid membranes demonstrate high thermal stability in the fuel cell operation temperature range (<100 °C). The addition of the MOF maintains the mechanical stability of the membranes (TS > 85 MPa in the Na^+^ form). Proton conductivity was analyzed using EIS, achieving the highest value with a 5 wt. % load of the HKUST-1. This value is lower than that observed for the HKUST-1/Nafion system. However, polarization and power density curves show a remarkably better performance of the hybrid membranes in comparison to both the pure SPES and the pure Nafion membranes.

## 1. Introduction

Electrochemical energy sources, such as fuel cells (FC), and energy storage devices (batteries and supercapacitors) are considered a good proposal to reduce worldwide CO_2_ emissions from fuel usage. According to the International Energy Agency, the key pillars of the decarbonization of the global energy system are energy efficiency, behavior change, electrification, renewable energy, hydrogen, and hydrogen-based fuels. Thus, sustained growth in demand for hydrogen and the introduction of cleaner technologies for its production allow hydrogen and hydrogen-based fuels to avoid CO_2_ emissions by up to 60 Gt in 2021–2050. This represents 6% of the total cumulative emission reductions. The development of hydrogen energy is due to the use of new nanostructured materials, for example, metal-organic frameworks, nanoparticles, carbon structures, and others [1,2,3,4]. Hydrogen fuel cells are clean conversion energy devices for a variety of applications, such as stationary energy sources, vehicles, and even portable devices [5,6,7]. Fuel cells transform the chemical energy retained in fuels for heating, water, and electric energy. Fuel cells have a high level of environmental safety. Wider use of FC will reduce pollution and the greenhouse effect.

There are many types of FCs. Proton-exchange membrane fuel cells (PEMFCs) are the most promising which comprise a cathode, an anode, and an electrolyte composed of a proton exchange membrane (PEM) [8,9]. The main role of the membranes in a PEMFC lies in providing canals for proton transferring to dissociate gas reactants and to isolate electrons [10]. An ideal membrane should have high proton conductivity and good mechanical and chemical stability. The level of membrane hydration directly affects their proton conductivity. Thus, these membranes must be hydrated. For this reason, the evaporation of water must occur at the same rate as it is produced. If water evaporates very quickly, the membrane dries out and the resistance across it increases.

Currently, the most popular membrane in PEMFCs is Nafion^®^, which is a perfluorosulfonic acid or PFSA membrane designed in the early 1970s by Dupont [11]. It has high ionic conductivity and chemical resistance. However, it displays some disadvantages, including low thermal and mechanical stability, high cost, and oxygen permeability [12].These drawbacks make it necessary to search for novel electrolytes based on different polymeric backbones [8], such as sulfonated poly(ether ether ketone)s (SPEEK) [13,14], sulfonated poly(ether sulfone) (SPES) [15], or sulfonated polyimides (SPI) [16,17]), which are characterized by their low cost and high chemical and thermal stabilities [18]. Polysulfone (PSU) has high chemical, thermal, and mechanical stability [19]. However, these polymeric backbones have lower ionic conductivity than Nafion^®^ (at low relative humidity (RH) values). Particularly, highly sulfonated PSU shows a high ability to absorb water (WU%) which leads to the deterioration of dimensional stability. The use of multiblock copolymers for the synthesis of PEMs could be considered an effective strategy to prepare highly sulfonated copolymers with improved conductivity, thus maintaining their dimensional stability [20]. In this scenario, the synthesis of hybrid membranes based on multiblock copolymers doped with hydrophilic inorganic fillers allows the achievement of high proton conductivities at high temperature to be able to compete with Nafion^®^.

Hybrid membranes containing metal-organic frameworks (MOFs) [21,22] dispersed in the polymeric matrix can be considered a good proposal to prepare polymeric electrolytes. MOFs show high specific surface area and large pore diameter, and they can increase the number of stored water molecules in the membrane and, consequently, increase the proton conductivity. For example, metal-organic frameworks comprising metal centers are interconnected by organic linkers to form a three-dimensional porous lattice [23,24,25,26]. Lattice parameters and chemical properties of these types of materials can also be specified by a combination of various structural elements. More than 72,000 variations of MOF structures are known [27,28,29]. Pore diameters can reach 98 Å [30] and occupy more than 50% of the volume. Therefore, the surface area varies from 1000 to 10,000 m^2^/g [31], and the density of a MOF can reach 0.13 g/m^3^ [32]. As a function of the structure, composition, and proposed modifications of MOFs, they can have unique sensory [33], sorption [34], catalytic [35], and other properties. Thus, they have a variety of applications in the industry for the separation and storage of liquids and gases (hydrogen and methane), for the catalysis of carbon dioxide, in medicine for targeted drug delivery, or in the field of green electrochemical energy. It has already been attempted to use a MOF structure for modifying a PEM Nafion^®^ [36]. In this work, a hybrid membrane was fabricated from super acid sulfated Zr-MOF (SZM) and Nafion^®^. The fuel cell performance of this membrane at low RH is higher than that observed for Nafion^®^. The Bronsted acid sites in the SZM networks keep more water molecules, which promotes proton transferring at low humidity. As a result of the use of an organometallic framework structure, the water-retaining properties and proton conductivity improve in comparison with commercial membranes such as Nafion^®^.

Other structures have also been used, for example, UiO-66 (Zirconium 1,4-dicarboxybenzene MOF) loaded with ionic liquids 1-butyl-3 methylimidazolium hydrogen sulfate (BMIm.HSO_4_), 1-butylimidazole hydrogen sulfate (BIm.HSO_4_) and 3-triethylammonium propane sulfone hydrosulfate (TEA-PSO_4_) was incorporated into the polymer of SPEEK at various concentrations [37]. The effect of the concentration of the ionic liquid encapsulated in UiO-66 was defined by the morphology and the thermal and chemical stability of the hybrid membranes. The addition of 7.5 wt. % of UiO-66 to SPEEK made it possible to obtain materials with higher proton conductivity, which made this mass ratio the best for the inclusion of ionic liquids. High concentrations of an ionic liquid produce agglomerates which decrease ionic conductivity. Based on the results of this study, it is determined that UiO-66 loaded with the ionic liquid TEA-PSO_4_ is a good material to use in green technologies.

Most MOFs are crystallized materials and cannot easily form a film, limiting their use. In another work [38], hybrid membranes based on polybenzimidazole (PBI) were prepared by loading post-synthetically modified (PSM) UiO-66-NH2 MOFs into a polybenzimidazole polymer of aryl ether (OPBI). The original membranes containing OPBI and MOF nanofillers were doped with phosphoric acid (PA) to form PEM. The use of thermostable and hydrophilic UiO-66 led to increased proton conductivity, higher retention capacity of PA, and higher resistance of the hybrid membrane against oxidative degradation compared to the original OPBI polymer. This work demonstrates the benefits of using rationally designed MOF UiO-66 as a nanofiller to fabricate OPBI membranes that can provide high proton conductivity in a wide temperature range of up to 160 °C.

One of the promising materials for this purpose is the metal-organic framework structure HKUST-1. This is a nanoporous crystalline structure which contains 1,3,5 benzenetricarboxylate (BTC) linkers and wheel-coordinated copper (Cu II) clusters [39]. Previously, this structure has already been used to modify Nafion^®^ [40], where the HKUST-1/Nafion membrane showed enhanced proton conductivity and high IEC values. However, the use of Nafion has disadvantages, such as poor thermomechanical stability and high oxygen permeability. As a result, this membrane is subjected to degradation in real devices. An alternative for it could be the employment of multiblock copolymers, such as a copolymer based on sulfonated polysulfone and polyphenylsulfones, which show high thermal stability at high temperatures (80 °C) [20].

In this work, sulfonated multiblock copolymers were prepared where polysulfone (PSU) and polyphenylsulfone (PPSU) segments were used at a ratio 5:5 (Figure S1). These copolymers were doped with the metal-organic framework structure HKUST-1 (Figure S2) to prepare the hybrid membranes. A complete characterization, including a morphological, thermal, mechanical, and electrochemical study, was performed. The fuel cell test was also carried out to evaluate the behavior of these hybrid membranes as a solid electrolyte.

## 2. Experimental Part

### 2.1. Materials and Reagents

HKUST-1 (Basolite C-300) was purchased from Sigma-Aldrich (Munich, Germany). N,N-dimethylacetamide (DMAc, 99.0%), and dimethyl sulfoxide-d_6_ (DMSO-d_6_, 99.9%) were purchased from Acros Organics (Geel, Belgium) and used as received. Sulfonated multiblock copolymers (SPES) were synthesized based on a previously described method [20] by polycondensation in a “one pot two-step synthesis” of commercial monomers. Trimethylsilyl chlorosulfonate (TMSCS) was used as a sulfonating agent to obtain SPES. A PSU unit:TMSCS molar ratio of 1:9 was used in the sulfonation reaction.

### 2.2. Membrane Preparation

Hybrid membranes were prepared by incorporation of the MOF HKUST-1 in the sulfonated multiblock copolymers of PSU and PPSU. The proportion of the PSU and PPSU blocks in the copolymer was 5:5. The sulfonated copolymer was prepared from a polymer:sulfonating agent molar ratio of 1:9. Three HKUST-1 loads (5, 10, and 20 wt. %) were used to prepare the hybrid membranes.

The membranes were obtained by casting. The SPES was dissolved (5 wt. %) in DMF with the addition of HKUST-1 powder in an amount of 5%, 10%, and 20% of the total mass. The solution was dried at 60 °C under vacuum for 48 h. The thickness of these membranes was 50 ± 10 µm. To replace sodium ions with protons, the membranes were treated with a 1 M HCl solution at 60 °C during 24 h.

### 2.3. Powder X-ray Diffraction (PXRD)

Powder X-ray diffraction (PXRD) patterns were collected from 5 to 45° (2θ) in continuous mode using an X’Pert Philips diffractometer with (θ-2θ) Bragg–Brentano geometry and equipped with a curved graphite monochromator. The measurements were obtained directly on the hybrid membrane, which was placed on a zero-diffraction plate made of a silicon single-crystal cut at a special orientation.

### 2.4. Field Emission Scanning Electron Microscopy (FE-SEM)

The morphology of the membranes was analyzed by using a FEI TENEO-LoVac FE-Scanning Electron Microscope. A voltage of 0.2–30 kV was used to carry out the measurements. The instrument was equipped with an EDAX TEAM™ EDS Analysis System. To evaluate a possible micro-phase separation of the hybrid membrane by the FE-SEM, protons of sulfonic groups were exchanged by Pb^2+^ ions [20]. The samples in the sodium form were treated with a 1 M of HCl solution to convert Na^+^ into H^+^. Deionized water was used to wash the membranes before being treated with a 1 M of Pb(NO_3_)_2_ solution during stirring for 48 h. Before doing these measurements, the samples were dried at 60 °C.

### 2.5. Contact Angle Measurements

To obtain information about the hydrophilicity of the membranes, surface contact angle measurements were conducted in a Dataphysics OCA15 plus goniometer with a SCA20 software (DataPhysics Instruments GmbH, Filderstadt, Germany). The membranes (SPES and SPES@HKUST-1) in the acidic form (H^+^) were used to carry out the assay. A sessile drop technique was employed to obtain the contact angle. A drop of water was positioned on the surface of a dry membrane, and the angle between the plane of the membrane and the drop was measured after 5 min. At least five measurements were performed on each membrane.

### 2.6. Water Uptake

Water uptake (WU%) of the membranes was analyzed by measuring the difference in weight before and after hydration. The membranes were dried at 60 °C under vacuum to determine the weight of the membranes in the dry form (*w_dry_*). Weight of the membranes in the wet form (*w_wet_*) was obtained by immersing the samples in deionized water for 24, 48, and 72 h. Before measurements, the surface of the membranes was dried with blotting paper. This parameter was obtained in triplicate. The parameters of WU% were evaluated for two temperatures (30 and 60 °C) by using Equation (1). The thickness of the hybrid membranes was around 50 µm.

(1) $$\mathrm{WU}\%=\frac{w_{wet}-w_{dry}}{w_{dry}}$$

### 2.7. Thermal Stability

The thermal stability of the membranes was analyzed by using a PerkinElmer Pyris STA 6000 instrument (Waltham, MA, USA). A sample of 7.5–10 mg was put in an Al_2_O_3_ pan and it was heated from 40 °C to 600 °C with the rate of 10 °C/min under nitrogen atmosphere. The onset decomposition temperature (*T*_OD_; temperature from which the weight loss begins) and the fastest decomposition temperature (*T*_FD_; temperature of the maximum in the weight loss rate) were determined from the derivative thermogravimetric (DTG) curve.

### 2.8. Mechanical Properties

A dynamo mechanical analyzer TA Instruments (DMA Q800) (New Castle, DE, USA) was used to carry out the dynamo-mechanical analysis of the hybrid membranes. The tensile mode was applied to provide measurements. The length, width, and thickness of the membranes were 12.0 mm, 2.5 mm, and 50 µm, respectively. All measurements were performed by using an initial static force equal to 0.15 N. Stress–strain tests were conducted at 30 °C using a ramp force of 0.3 N min-1 to achieve 18.0 N in the controlled force mode. A temperature range from 30 to 250 °C was used to carry out the DMTA analysis with a rate of 2 °C per minute at 1 Hz. The membranes in the Na^+^ and H^+^ forms were evaluated. The first ones were dried at 60 °C under vacuum for 48 h before performing the measurements. However, the second ones were treated with a 1 M of HCl solution (during 24 h at 60 °C) to convert the membranes in the H^+^ form. Each test was performed in triplicate.

### 2.9. Ion-Exchange Capacity

An acid–base titration was used to determine the ion-exchange capacity (IEC) of the hybrid membranes. The samples in the sodium form were treated with a 1 M of HCl solution (24 h, 60 °C) to obtain the proton form. A 2 M of NaCl solution was used to treat this membrane and replace H^+^ with Na^+^. The solution in contact with the membrane was titrated with a 0.01 M of NaOH solution which was normalized with potassium hydrogen phthalate and phenolphthalein as an indicator before its use. The titration was performed in triplicate. The following equation was used to calculate the IEC:

(2) $$\mathrm{IEC}=\frac{V_{NaOH}\times \left[NaOH\right]}{w_{dry}}$$

where *V_NaOH_* and [*NaOH*] are the volume and the concentration of NaOH, respectively, and *w_dry_* is the weight of the dry membranes.

### 2.10. Membrane Proton Conductivity

Ex situ proton conductivity measurements were performed by means of electrochemical impedance spectroscopy (EIS). An impedance analyzer (Solartron 1260) with an electrochemical interface (Solartron 1287) was employed. A frequency range from 10^−1^ Hz to 1 MHz with a voltage amplitude of 0.01 V was applied to carry out the electrochemical measurements. The samples were analyzed in the temperature range from 40 °C to 90 °C and a RH of 90%. Both parameters, temperature and RH, were controlled in a Binder KMF 115 (E5.2) constant climate chamber. Compacted powder or membrane was placed between two gold ion-blocking electrodes. The resistance values related to the pristine membrane, the MOF compacted powder, or the hybrid membranes was determined from the intercept point with the real axis in the Nyquist plot at high frequencies. The proton conductivity (σ in S cm^−1^) was calculated using the following equation:

(3) $$\sigma =\frac{L}{R\times A}$$

where *L* and *A* are the thickness and the area of the membranes, respectively, and *R* is the ohmic resistance. A Z-view analysis impedance software (Scribner Associates, Inc., Southern Pines, NC, USA) was used to analyze the obtained data.

### 2.11. Fuel Cell Test

Membrane electrode assembly (MEA) with an active area of 5 cm^2^ was created by placing the membrane between two identical electrodes (70 wt. % Pt, Paxitech) with a Pt loading of 0.5 mg Pt cm^−2^. 

Performance tests were conducted in a Scribner 850e multi-range fuel cell test system at 100% RH, atmospheric pressure, and cell temperature from 50 to 80 °C. H_2_ and O_2_ were used as the fuel and comburent, respectively, at a flow rate of 200 mL/min.

Electrochemical measurements were analyzed by means of an experimental ElectroChem Inc. single cell hardware.

In situ through-plane proton conductivity of the membranes was determined by means of EIS using a potentiostat Autolab PGStat30 appointed with an FRA module in a temperature range from 50 °C to 80 °C at 100% RH. The frequency diapason applied was in the range from 10 kHz to 1 Hz and the amplitude of the sinusoidal signal was 10 mV. Humidified hydrogen (SHE, anode) and nitrogen (cathode) at a flow rate of 200 mL/min were continuously fed to the cell. Finally, the pseudo-activation energies (*E*_a_^VTF^) were calculated according to the Vogel–Tammann–Fulcher (VTF) Equation (4), a widely used approximation for non-Arrhenius polymer ionic conductors:

(4) $$\sigma =\sigma_{o}\;exp\left(\frac{-E_{a}^{\mathrm{VTF}})}{K\left(T-T_{0}\right)}\right)$$

where *σ*_0_ is the pre-exponential factor; *T* is the absolute temperature; *K* is the Boltzmann constant; *E*_a_^VTF^ is the pseudo-activation energy; and *T*_0_, when considering the polymers, is the glass transition temperature at which the “free” volume disappears or at which the configuration free entropy becomes zero. In this case, *T*_0_ could also be associated with the temperature at which molecular water motions cease [41].

## 3. Results and Discussion

### 3.1. Characterization of Hybrid Membranes: Powder X-ray Diffraction

**Figure 1:**
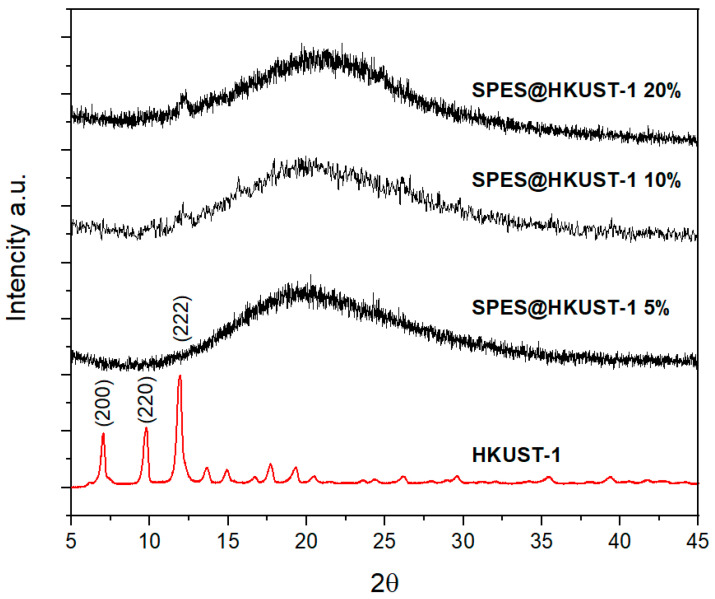
Powder X-ray diffraction of the SPES@HKUST-1 membranes with different amount of HKUST-1 (5, 10, and 20%) and the pristine MOF HKUST-1.

In order to determine the presence of MOF crystallites in the membranes, X-ray powder diffraction experiments were performed. Figure 1 shows the XRD patterns of the hybrid membranes (with MOF loads of 5, 10, and 20%) and the pristine MOF HKUST-1. All of them present a very broad peaks centered around 2θ = 20°, characteristic of the polymeric matrix. The XRD pattern of the pure HKUST-1 shows more intense and defined peaks due to its higher crystallinity, as reported elsewhere [42]. The main diffraction peak of the MOF, which is associated with the (222) plane, emerges as a small peak in the XRD patterns of the more loaded membranes but does not appear for the low-concentration (5%) sample.

### 3.2. Optimization of the Amount of Filler

**Figure 2:**
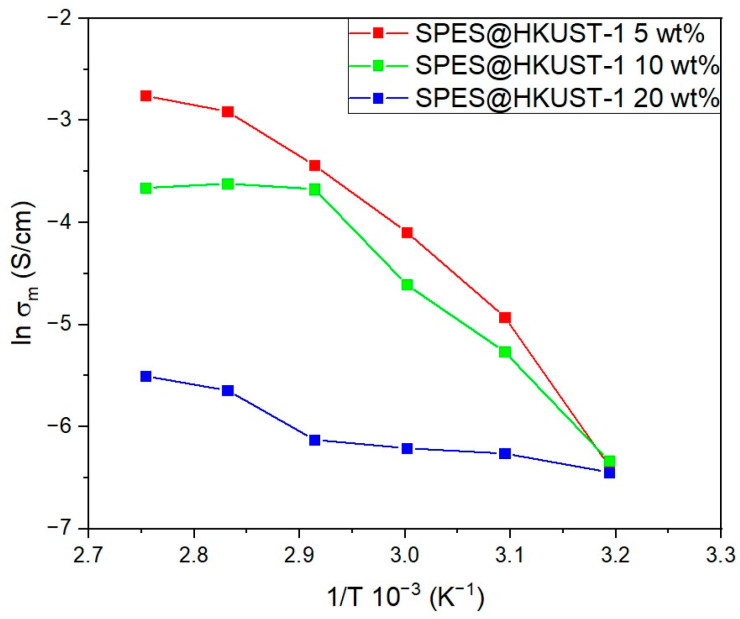
Ex situ proton conductivity of the SPES@HKUST-1 membranes with MOF HKUST-1 loading of 5, 10, and 20 wt. %, as a function of temperature at 90% RH.

With the aim of finding the optimal HKUST-1 load used in the preparation of these hybrid membranes, ex situ ionic conductivity measurements were conducted. Ionic conductivity was chosen as a control key parameter for the optimization of the required amount of MOF, since polymeric electrolytes must have high proton conductivity to achieve high fuel cell performance. Figure 2 represents the evolution of ex situ proton conductivity of the hybrid membranes as a function of temperature (40–90 °C) at 90% RH for various HKUST-1 loads (5, 10, and 20 wt. %).

The obtained results reveal that the membrane doped with 5 wt. % of MOF shows the highest ionic conductivity in all temperature range. Thus, the proton conductivity of this membrane at the operating temperature of fuel cell (90 °C) is considerably higher (63.4 mS cm^−1^) than those obtained for higher percentages of MOF (25.8 and 4.1 mS cm^−1^ for 10 and 20 wt. %, respectively). In addition, the fact that ionic conductivity does not decrease with temperature at high temperatures is a good indicator of the high stability of this material in the temperature range for PEMFC operation. Therefore, in view of these results, the membrane doped with 5 wt. % of MOF was chosen to be studied in this work. A complete characterization of this membrane, which includes a morphological, thermal, mechanical, and electrochemical study, is discussed besides their behavior as a solid electrolyte in a single fuel cell.

### 3.3. Microstructural Characterization

**Figure 3:**
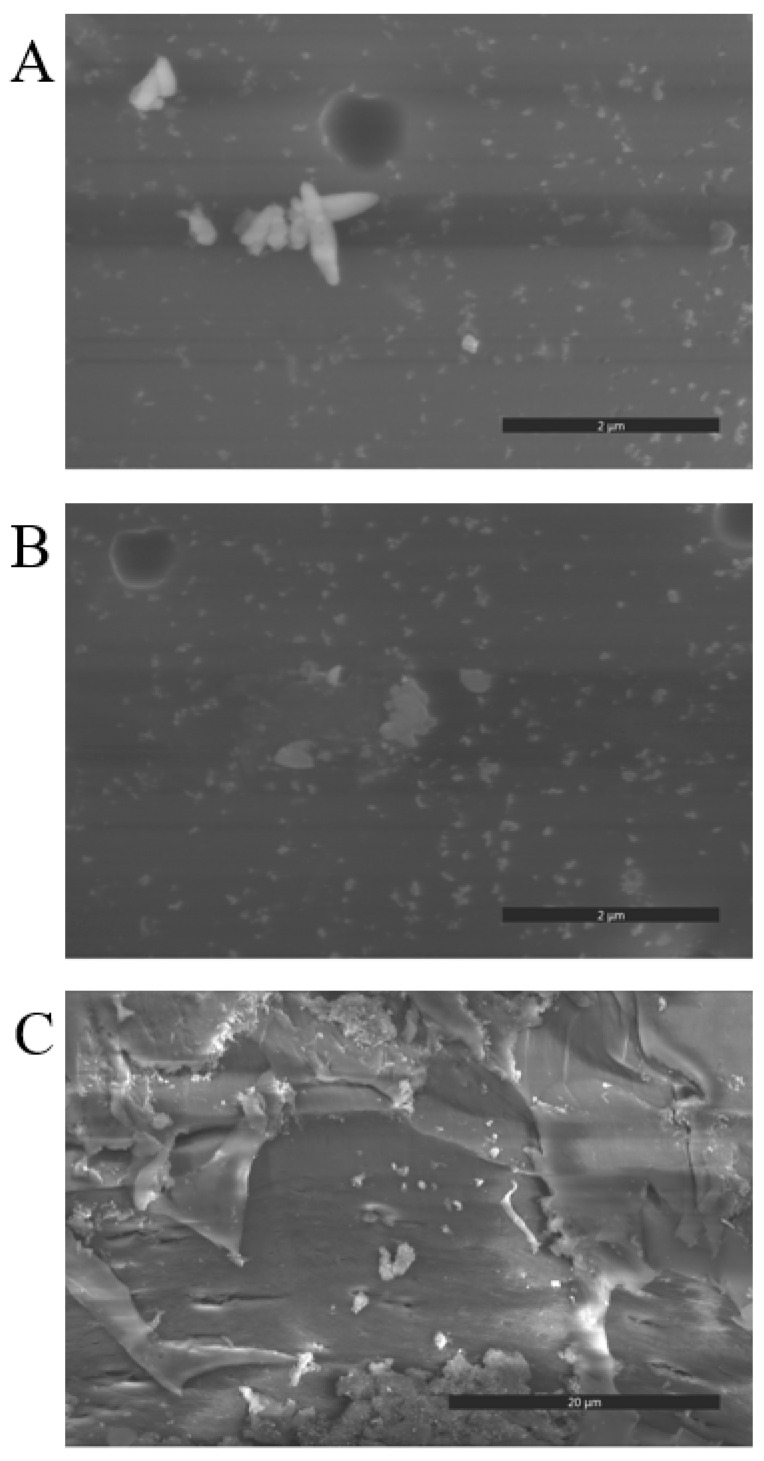
SEM images of both surfaces (**A**) (top) and (**B**) (bottom) of the SPES@HKUST-1 membrane (5 wt. % of HKUST-1), and its cross-section (**C**).

The presence and the distribution of HKUST-1 crystallites in the hybrid membrane are analyzed by means of scanning electron microscopy. Figure 3 shows the SEM images of the SPES@HKUST-1 5% hybrid membrane on both surface sides (Figure 3A,B), and its cross-section (Figure 3C). HKUST-1 crystallites with a size of about 500 nm and even lower are identified in all images. These images reveal that the metal-organic framework particles are quite well distributed throughout the membrane. Furthermore, there is no accumulation of MOF particles on one surface side, and this is not easily achieved when membranes are prepared by casting.

**Figure 4:**
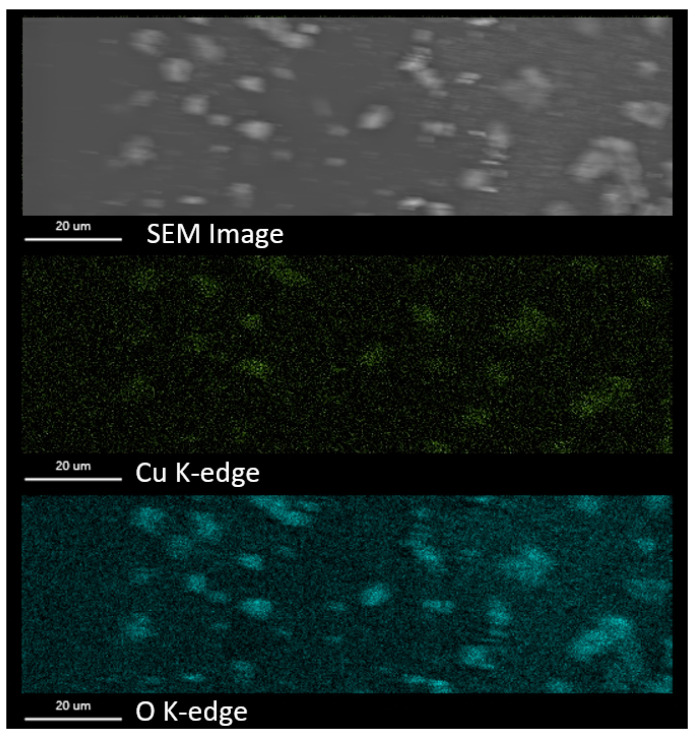
EDX mapping of the SPES@HKUST-1 membrane (5 wt. % of HKUST-1).

To confirm the homogeneous distribution of HKUST-1 crystallites in the membrane, EDS mapping is obtained (Figure 4). Thus, it can be seen that the highest concentration of copper is observed at the locations of the crystallites. This fact is attributed to the copper clusters which are an inorganic structural element of the HKUST-1. In addition, in the same regions, there is a large amount of oxygen, which could come from the organic component of the MOF, and probably from the water molecules that are located on the surface of the HKUST-1.

### 3.4. Water Uptake, IEC, and Contact Angle

The water absorption capacity of the PEM is a key property for their operation in a low temperature FC, since it affects mainly fundamental parameters, such as proton conductivity, and mechanical properties. The WU% values at two different temperatures for both pristine SPES and hybrid membranes are determined (Table 1). At 30 °C, the WU% of the pristine membrane is 24%, and this value is practically doubled (44%) by incorporating the MOF in the polymer matrix. The effect that the MOF has on WU% is considerably higher at 60 °C, i.e., WU% increases from 31 to 74%. The ability of the HKUST-1 to absorb water is also observed in other hybrid membranes based on the Nafion modified with MOF-808, where the maximum WU% obtained is 20% [36], or the SPEEK modified with Zr-MOF, which WU% is 70% [37].

The high capacity of the hybrid membranes to absorb water is closely related to their hydrophilicity, which was evaluated by means of contact angle measurements. Thus, the SPES@HKUST-1 membrane displays a lower contact angle (70.6°) than the pristine SPES membrane (80.3°), revealing a higher hydrophilicity of the hybrid membrane which is attributed to the presence of the HKUST-1.

At the same time, the IEC increases from 1.62 to 1.93 (m_equiv_ H^+^ g^−1^) when the MOF is incorporated in the polymer matrix. This trend is associated with an increase in water absorbed by the hybrid membrane through the MOF structure. Therefore, in view of these results, the presence of the HKUST-1 in the membrane favors the exchange of counterions.

### 3.5. Thermal Characterization

**Figure 5:**
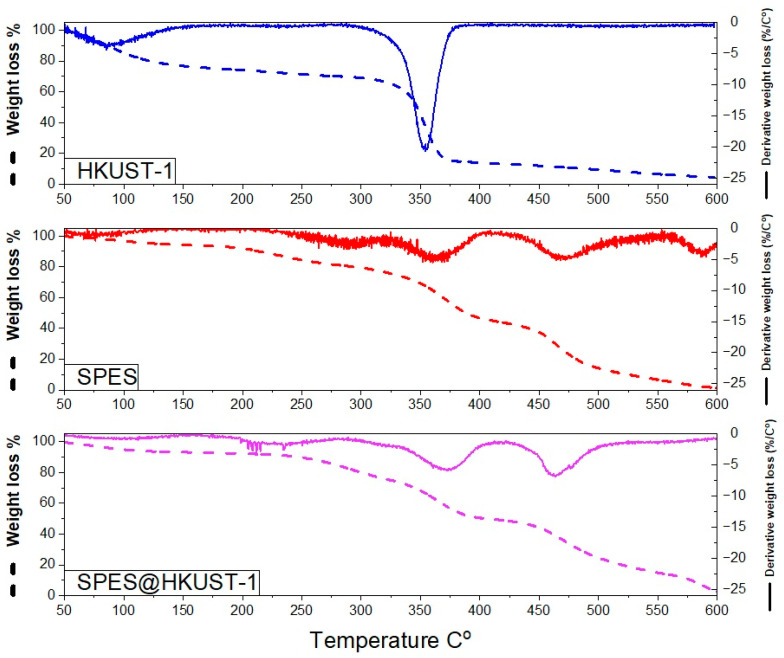
TGA and DTG curves for the powder of the HKUST-1, the SPES membranes, and the SPES@HKUST-1 membranes under air atmosphere.

TGA and DTG curves for the metal-organic framework, pristine SPES, and SPES@HKUST-1 hybrid membranes are shown in Figure 5.

The thermogravimetric curve obtained for the metal-organic framework HKUST-1 is in accordance with that reported by Fernandez-Catala et al. [44]. A first weight loss of approximately 20% at a temperature lower than 150 °C is associated with the elimination of water molecules retained in the MOF structure. The second weight loss appears in the temperature range from 300 to 350 °C (T_OD_~288 °C and T_FD_~355 °C), and it can be observed that the HKUST-1 is stable until ~325 °C, after which an almost complete degradation of the crystal structure is observed.

For the pristine SPES membranes, several weight losses are observed. The first loss at 150 °C corresponds to water removal, whereas the second one at higher temperatures from 200 to 350 °C (T_OD_~225 °C and T_FD_~285 °C) is attributed to the degradation of sulfonic groups. From 350 °C onwards, the polymer backbones are decomposed.

A similar thermal behavior is observed for the hybrid membrane SPES@HKUST-1. The first weight loss below 150 °C is associated with the removal of water from both the pores of the MOF structure and SPES. The second weight loss observed between 200 and 350 °C (T_OD_ and T_FD_ could not be accurately determined from the DTG curve) is due to the decomposition of sulfonic groups. Finally, at higher temperatures, the thermal decomposition of the polymer backbone occurs.

It should be noted that the hybrid membrane maintains a high thermal stability at temperature lower than 200 °C, which allows its use as a solid electrolyte in a conventional hydrogen fuel cell, where the working temperature is up to 90 °C.

### 3.6. Mechanical Properties

The thermomechanical properties of the membranes limit their performance and durability in the fuel cell. Thus, high tensile strength (TS) and ductility over a wide range of temperature and humidity allow ionomers to maintain the shape of thin and non-fragile films during the fuel cell operation. The membranes must be adapted to changes under the operating conditions while maintaining their mechanical stability. To evaluate their behavior, both stress–strain tests and dynamo mechanical tests were carried out.

**Figure 6:**
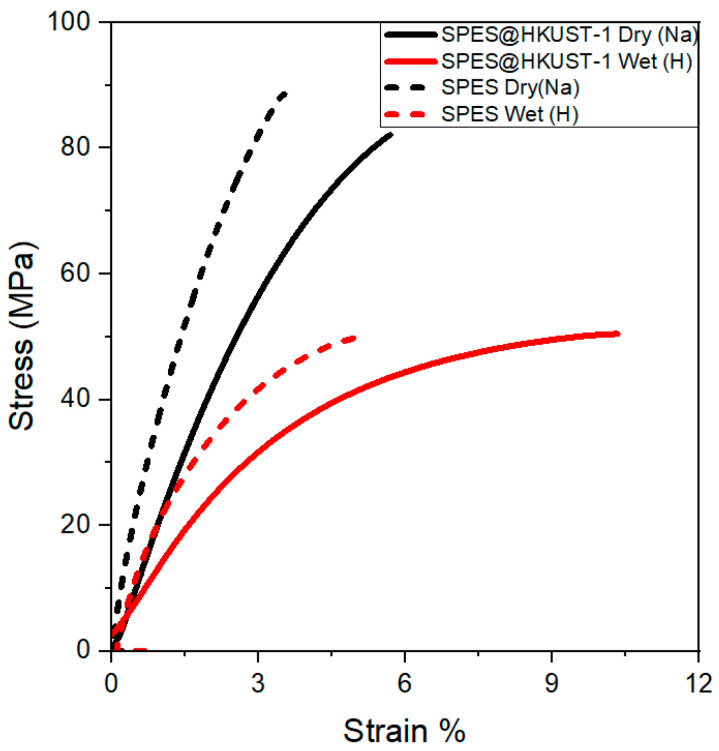
Tensile stress–strain curves for the SPES@HKUST-1 and pristine SPES membranes at 30 °C.

Figure 6 shows the tensile stress–strain curves of the pristine SPES and SPES@HKUST-1 hybrid membranes. The TS values obtained are displayed in Table 1. As it is well known, the mechanical properties of proton exchange membranes strongly depend on their hydration degree. Thus, the stress–strain tests were carried out on the membranes in both Na^+^ (dry) and H^+^ (wet) forms. Mainly, the membranes in sodium form show higher tensile strength and, above all, higher stiffness and lower ductility (low elongation at break) than the wet membranes. For example, the stiffness of the SPES@HKUST-1 membrane in dry form is around 11,584 N m^−1^ in comparison to 5878 N m^−1^ in the wet form. Comparably, the TS decreases from 88 ± 4 to 49 ± 5 MPa when the membranes are immersed in water. However, the elongation at break increases from 5.7% to 11.1% in the wet form. The pristine membrane exhibits a similar mechanical behavior. Therefore, these parameters are strongly related to the water adsorbed and water molecules seem to act as plasticizers [45,46], thus decreasing the TS values. To conclude, in all cases, the TS is higher than those observed for the commercial membrane Nafion 112 [47], which shows a value around 19 MPa when measured under similar experimental conditions.

The more hydrophilic character of the hybrid membrane, compared to the pristine one, promotes greater water absorption. However, a significant loss of mechanical properties is not evidenced.

**Figure 7:**
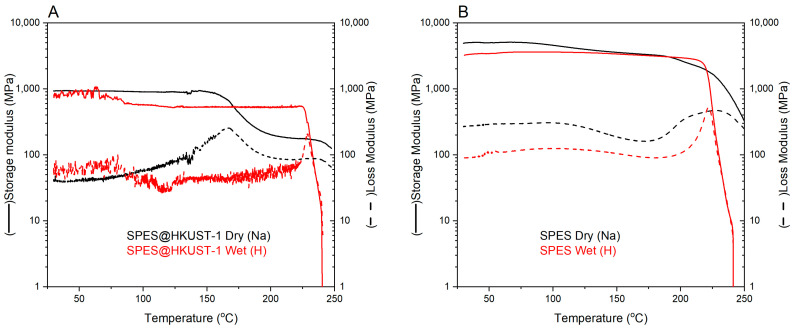
DMA curves for (**A**) the SPES@HKUST-1 membrane and (**B**) the pristine SPES membrane.

To analyze the thermo-mechanical behavior of the pristine and hybrid membranes in sodium and proton forms, the evolution of both storage and loss modulus with temperature is studied (Figure 7). The relaxation temperature (*T*_α_) is obtained from a decrease in curve of storage modulus which, in turn, corresponds to the maximum of the loss modulus curve. The storage modulus at 50 °C (E′_50°C_) and the *T*_α_ values are shown in Table 1.

The addition of the HKUST-1 to the membrane exerts a great influence on E′_50°C_. Thus, the hybrid membranes show lower values than the pristine ones. This can be justified by the fact that [23] the specific surface area and sorption capacity of the HKUST-1 allow water molecules to be in the pores of the structure even in the sodium form of the membrane. Undoubtedly, the presence of MOF crystallites also contributes to the modulus of elasticity of the material. Despite the good distribution of the HKUST-1, the presence of minimal defects in the integrity of the membrane cannot be excluded, as well as the interaction of the hydrogen atoms present on the linker of the metal-organic framework with the sulfonic groups of the membrane.

In the transition to the H^+^ form (from the Na^+^ form), the E′_50°C_ of the SPES@HKUST-1 membrane does not change significantly, in contrast to the pure membrane. The interaction between the HKUST-1 and sulfonic groups seems to be enough strong to observe a significant difference in this parameter when Na^+^ is replaced by H^+^.

The relaxation temperature decreases from 230 °C to 166 °C with the addition of the HKUST-1. At the same time, the *T*_α_ of the modified membrane increases and becomes almost equal to the pure membrane in the acidic form. In the sodium form, Na^+^ is located on sulfonic groups, while H^+^ are located on both sulfonic groups and carbon linkers of the MOF, and water molecules are also in the pores. In the conversion into the acidic form, the interaction between the sulfonic groups of the membrane and the HKUST-1 changes from Na-H to H-H which, in turn, can affect the properties of the membrane. However, these temperatures are too high for PEMFCs and they do not limit the effectiveness of these membranes in the fuel cell.

### 3.7. Ex Situ Proton Conductivity

The ex situ proton conductivity of the different materials (compacted pristine HKUST powder, pristine SPES membrane, and hybrid SPES@HKUST-1 membrane) is determined by the EIS. The Nyquist plots for the compacted powder of the HKUST-1, the pristine SPES membrane, and the SPES@HKUST-1 hybrid membrane are shown in the Supplementary Information (Figures S3, S4, and S5, respectively).

**Figure 8:**
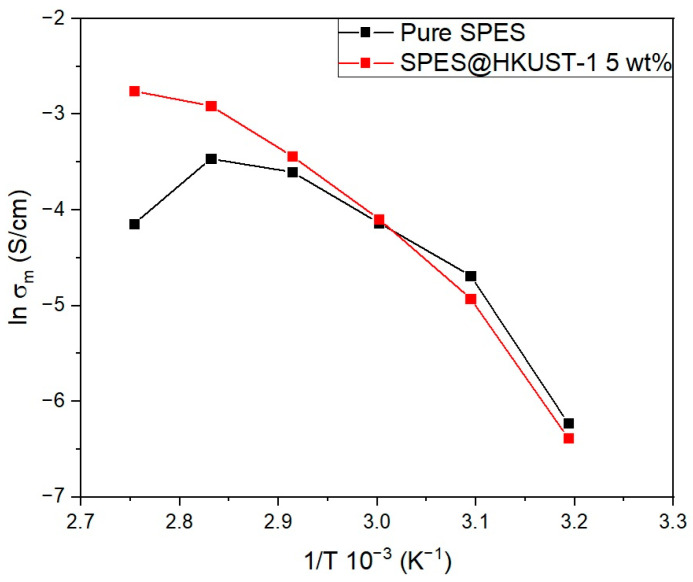
Proton conductivity of the pristine SPES membrane and the SPES@HKUST-1 with HKUST-1 loading of 5 wt. %, as a function of temperature at 90% RH.

The evolution of proton conductivity with temperature at a relative humidity of 90% for the pristine membrane and the hybrid membrane is shown in Figure 8. The contribution of the MOF to the ionic conductivity of the hybrid membrane is analyzed from the data obtained for both the pristine and hybrid membranes. The SPES@HKUST-1 and MOF-free membranes show similar proton conductivity at low temperatures (40–70 °C). However, when paying attention to temperatures close to the operating conditions of the fuel cell (80–90 °C), it can be noted that the conductivity of the SPES@HKUST-1 is much higher (63.4 mS cm^−1^ at 90 °C and 90% RH) than the conductivity of a pure SPES (15.8 mS cm^−1^). These data are in good agreement with the WU% and IEC values (see Table 1), given that more water molecules and mobile protons are in the hybrid membrane, and, therefore, the higher ionic conductivity. The addition of the HKUST-1 that allows the membrane to retain water molecules at higher temperatures explains their high conductivity at 90 °C. A similar behavior has been reported in the literature for sulfonated polysulfone doped with titanium dioxide [48].

In the same line, the membrane based on Nafion doped with the HKUST-1 [40] exhibits higher conductivity than pristine polymer. In this hybrid membrane, the water uptake decreases with MOF and the acid groups (H_3_PO_4_) incorporated into the membrane are responsible for the conduction of protons.

As shown in Figure 8, proton conductivity does not follow the Arrhenius equation, and therefore the Vogel–Tammann–Fulcher equation (Equation (4), see experimental section) is used to determine the pseudo-activation energy of the process. Thus, *E*_a_^VTF^ is estimated for both the hybrid and pristine membranes as shown in Figure S6 and Table S1. Thereby, *E*_a_^VTF^ value is 0.034 and 0.036 eV for the pristine SPES and hybrid SPES@HKUST-1 membranes, respectively. These values are not significantly different, which would indicate that both membranes follow a similar reaction mechanism for the proton transport. According to the values of the activation energy obtained, the transfer of ions seems to be carried out through the Grotthus mechanism.

### 3.8. Electrochemical Characterization of the MEA

**Figure 9:**
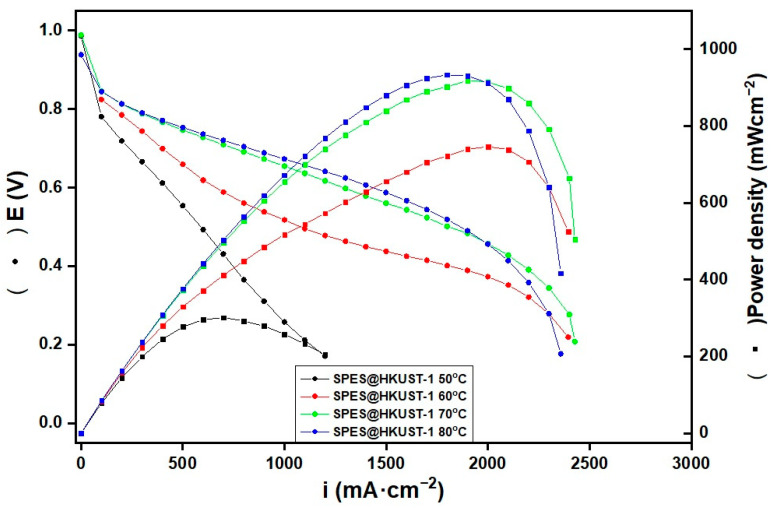
Polarization and (B) power density curves of the SPES@HKUST-1 membrane at 50, 60, 70, and 80 °C and 100% RH.

The behavior of the SPES@HKUST-1 membrane was tested in a single fuel cell. In order to compare with previous tests of pristine membrane SPES [23], the measurements were obtained under the same experimental conditions. The MEA with 5 cm^2^ active area was evaluated in a hydrogen single cell in the temperature range from 50 °C to 80 °C, under 100% RH and atmospheric pressure. Figure 9 shows the polarization (A) and power density (B) curves of this membrane as a function of temperature. As shown in this figure, the best performance is achieved at high temperatures (70 °C and 80 °C) where the maximum power densities are ~900 mWcm^−2^ and a maximum current density of nearly 2400 mA cm^−2^ is reached. These results are very promising if they are compared with those performed by commercial Nafion membranes with similar thickness and measured under the same experimental conditions. Nafion 112 achieves a maximum power density of 729 mW cm^−2^ which corresponds to a maximum current density of 2400 mA cm^−2^, while Nafion 117 reaches a maximum power density of 310 mW cm^−2^ with a maximum current density of 999 mA cm^−2^. On the other hand, it is important to point up that the pure polymer SPES membrane shows a maximum power density of only 400 mW cm^−2^ for a current density of 1100 mA cm^−2^ [20], which represents less than half of the values reached in the case of the SPES@HKUST-1 membrane.

Table 2 shows the membrane resistance and proton conductivity values of the pristine [20] and SPES@HKUST membranes obtained by in situ through-plane measurements. The atmospheric pressure and a wide temperature range with fully humidified gases (100% RH) were adjusted to carry out the measurements. It can be noted that the ionic conductivity of the hybrid membrane increases when increasing temperature in a similar way as that observed in the ex situ conductivity measurements presented above (Figure 9), while in the case of SPES membrane, the proton conductivity value decreases at 80 °C. The better water absorption properties and higher hydrophilicity of the hybrid membranes due to the addition of the MOF as well as the higher ion exchange capacity would be responsible for the improved electrochemical behavior.

## 4. Conclusions

In this study, hybrid membranes based on sulfonated multiblock copolymers of PSU and PPSU doped with the HKUST-1 are successfully synthesized and characterized. The optimal powder loading (5 wt. %) to be incorporated into the polymeric matrix is selected by means of ex situ proton conductivity measurements. The FE-SEM micrographs and EDX mapping reveal a homogeneous distribution of HKUST-1 crystallites into the polymeric membranes. The presence of MOF particles favors water retention mainly at high temperatures; thus, the highest proton conductivity is at temperatures close to 90 °C. Furthermore, this improvement in the transport properties of the hybrid membranes takes place without reducing their mechanical stability, which means they maintain their working life.

To determine the contribution of the HKUST-1 to the behavior of the proton-exchange membrane SPES, the experimental data obtained for the hybrid membrane were compared with previously published data for the pure membrane. The hybrid membrane shows (i) better water absorption properties (44% and 74% opposite to 24% and 31% for 30 and 60 °C, respectively); (ii) higher ionic exchange capacity (1.93 opposite to 1.62 m_equiv_ H^+^ g^−1^); (iii) similar mechanical and thermal properties; and (iv) higher ex situ ionic conductivity at high values of relative humidity (90%) and temperature (90 °C), specifically 63.4 mS cm^−1^ for a modified membrane in contrast to 15.8 mS cm^−1^ for a pure membrane. A similar behavior is also observed in the HKUST-Nafion system [40] where the presence of this MOF enhances the proton conductivity of the membrane. In addition, the hybrid membrane shows higher values of in situ through-plane ionic conductivity as measured in a hydrogen single fuel cell at 80 °C than the pure SPES. Polarization and power density tests also reveal a remarkably better performance of the hybrid membrane. All the data obtained perfectly correlate with each other and allow us to conclude that the use of the SPES@HKUST-1 hybrid membranes makes it possible to approximately double the efficiency of the fuel cell test, while not impairing the stability of its operation

## Figures and Tables

**Table 1:** Characteristic parameters of the pristine SPES and hybrid SPES@HKUST-1 membranes.

Name	WU%		IEC (m_equiv_ H^+^ g^−g^)	Contact Angle (°)	*TS* (MPa)		E’_50C_ (MPa)		*T_αDMTA_* (°C)	
	30 °C	60 °C			Dry Na^+^	Wet H^+^	Dry Na^+^	Wet H^+^	Dry Na^+^	Wet H^+^
SPES	24 ± 5	31 ± 4	1.62	80.3	87 ± 8	56 ± 8	5004	3424	230	223
SPES@HKUST-1	44 ± 3	74 ± 3	1.93	70.6	88 ± 4	49 ± 5	930	922	166	227
PSU [43]	-	~20%	0.53	-	-	32.4	-	557	-	-

**Table 2:** In situ ionic conductivity and maximum power density values for the SPES and SPES@HKUST-1 membranes as a function of temperature.

*T* (°C)	SPES [20]			SPES@HKUST-1		
	R·10^2^ (Ω)	σ (mS·cm^−1^)	Power Density (mWcm^−2^)	R·10^2^ (Ω)	σ (mS·cm^−1^)	Power Density (mWcm^−2^)
50	6.00	19.7	251	6.36	12.2	301
60	4.58	25.8	340	3.92	19.8	746
70	3.47	34.1	394	2.51	30.9	918
80	3.97	29.8	403	2.47	31.4	933

## Data Availability

Data will be made available upon request. The raw/processed data required to reproduce these findings cannot be shared at this time as the data also form part of an ongoing study.

## Supplementary Materials

The following supporting information can be downloaded at https://www.mdpi.com/article/10.3390/polym15020323/s1, Figure S1: Metal-organic framework structure HKUST-1; Figure S2: Sulfonated multiblock copolymer (SPES); Figure S3: Impedance Nyquist plot of the metal-organic structure HKUST-1 at different temperatures; Figure S4: Simplified equivalent circuit for the conducting hybrid membrane sandwiched between two electrodes. R_cable_ and L_cable_, represent the impedance and inductance of cables and the empty cell, respectively. CPE_dl_ (constant phase element) represents the contribution of the membrane/electrode interfaces. R_b_ and C_b_ represent the bulk membrane resistance and capacitance, respectively. Impedance Nyquist plot of the pristine SPES membrane at different temperatures; Figure S5: Typical Nyquist plots for the SPES@HKUST-1 membrane; Figure S6: VTF fitting of the conductivity data from (A) the pristine SPES membrane and (B) the hybrid SPES@HKUST-1 membrane; Table S1: Parameters of the VTF equation obtained by fitting the conductivity data.
