# Supplementary material for: Using Metal-Organic Framework HKUST-1 for the Preparation of High-Conductive Hybrid Membranes Based on Multiblock Copolymers for Fuel Cells
Source: Polymers (Basel). 2023 Jan 8;15(2):323. doi: 10.3390/polym15020323 (PMC9862821; doi:10.3390/polym15020323)
Supplement: Supplementary file 1 [file polymers-15-00323-s001.zip › polymers-2134841-supplementary.pdf]

SUPLEMENTARY MATERIAL

Using Metal-Organic Framework HKUST-1 for the Preparation of High-Conductive Hybrid Membranes Based on Multiblock Copolymers for Fuel Cells

Ivan Gorban [1,2], Nieves Ureña [2], María Teresa Pérez-Prior [2,*], Alejandro Várez [2], Belén Levenfeld [2], Carmen del Río [3] and Mikhail Soldatov [1]

1 The Smart Materials Research Institute, Southern Federal University, Sladkova Street 178/24, 344090 Rostov-on-Don, Russia
2 Departamento de Ciencia e Ingeniería de Materiales e Ingeniería Química, IAAB, Universidad Carlos III de Madrid, Avda. de la Universidad, 30, Leganés, 28911 Madrid, Spain
3 Instituto de Ciencia y Tecnología de Polímeros (ICTP-CSIC), C/Juan de la Cierva, 3, 28006 Madrid, Spain
* Correspondence: maperezp@ing.uc3m.es; Tel.: +34-916249849

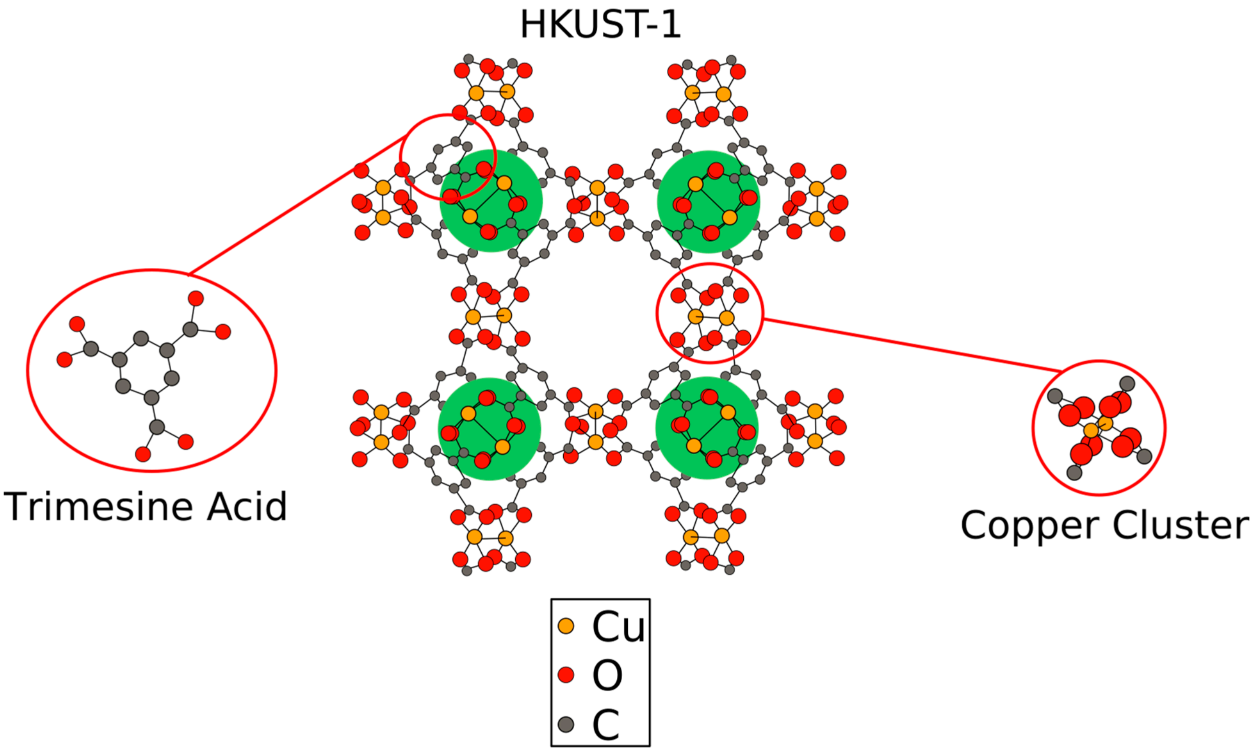

Figure S1 Metal-organic framework structure HKUST-1.

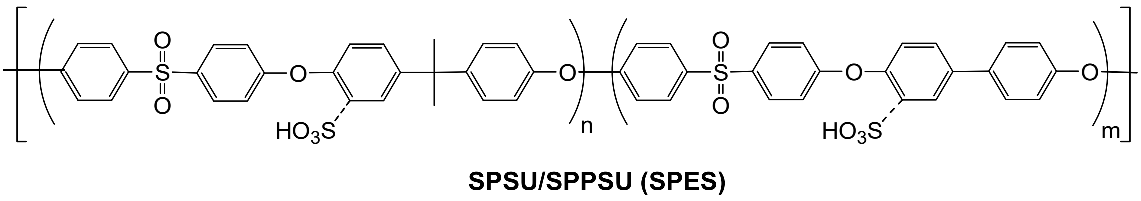

Figure S2. Sulfonated Multiblock Copolymer (SPES).

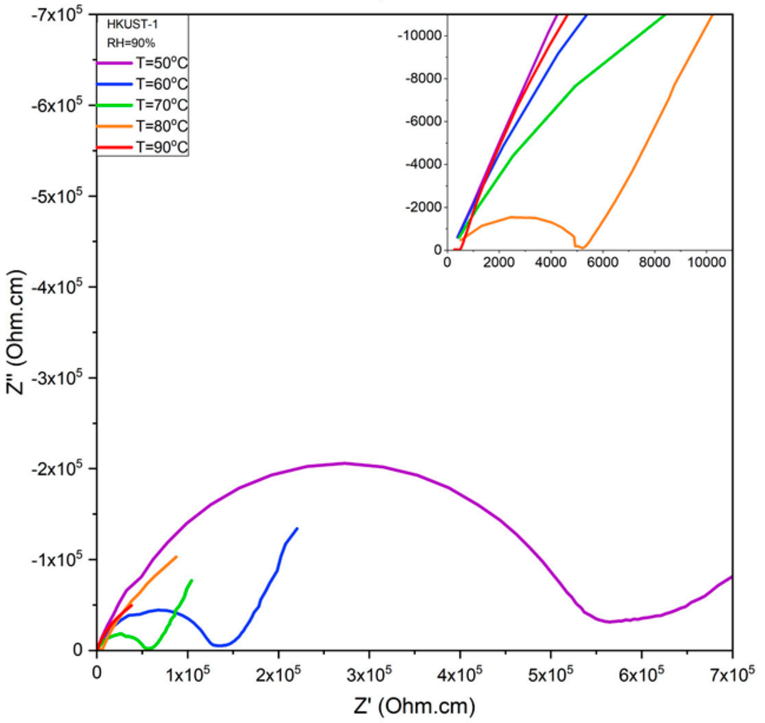

Figure S3. Impedance Nyquist plot of metal-organic structure HKUST-1 at different temperatures.

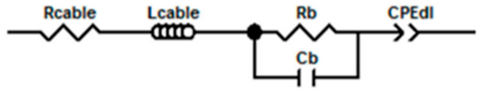

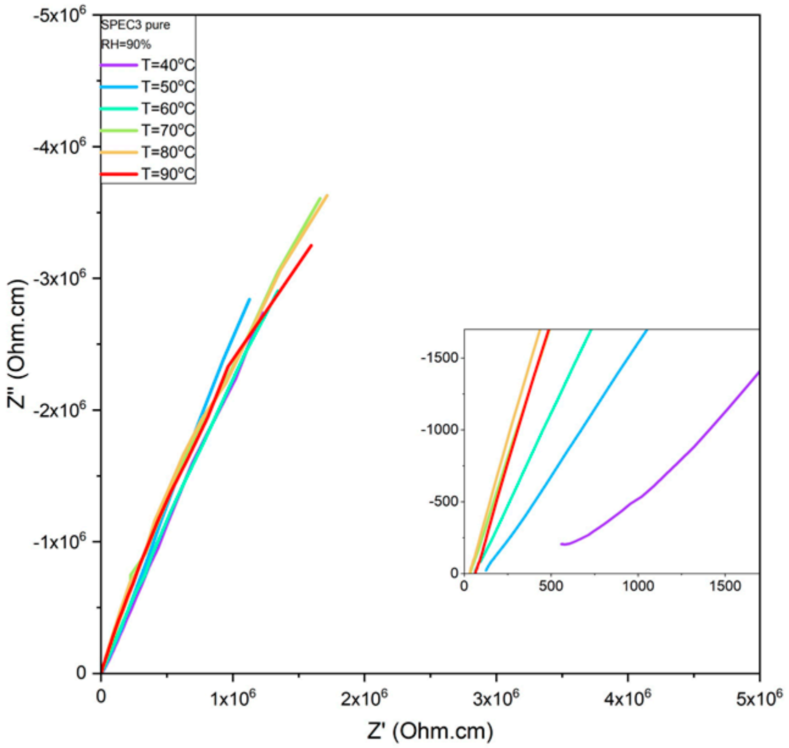

Figure S4. Simplified equivalent circuit for the conducting hybrid membrane sandwiched between two electrodes. R_{cable} and L_{cable}, represent the impedance and

inductance of cables and the empty cell, respectively. CPE_{dl} (constant phase element) represents the contribution of the membrane/electrode interfaces. R_b and C_b represent the bulk membrane resistance and capacitance, respectively. Impedance Nyquist plot of pristine SPES membrane at different temperatures.

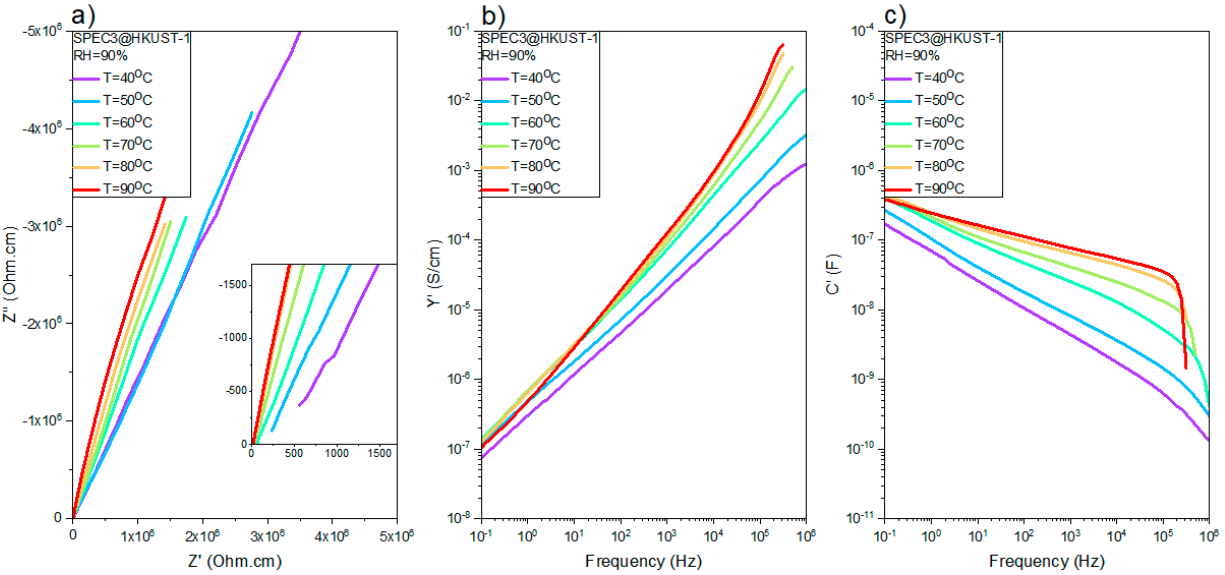

Figure S5. Typical Nyquist plots for SPES@HKUST-1 membrane.

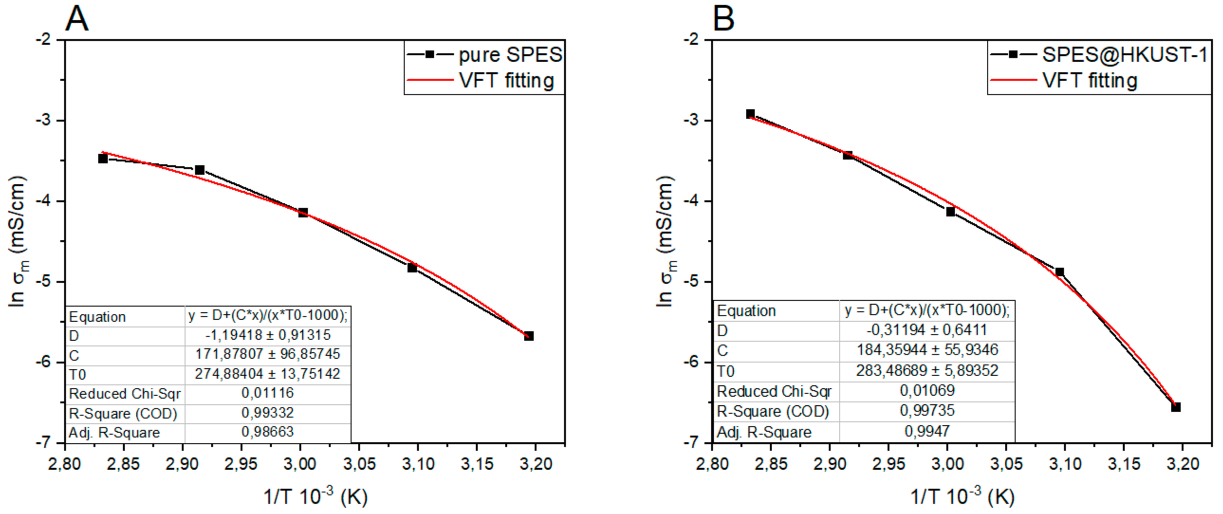

Figure S6. VTF fitting of the conductivity data from A) pristine SPES membrane and B) hybrid SPES@HKUST-1 membrane.

Table S1. Parameters of the VTF equation obtained by fitting the conductivity data

**VFT parameters**	Pure SPES	SPES@HKUST-1
$\boldsymbol{T_0}$ **(K)**	274	283
$\boldsymbol{E_a^{VFT}}$ **eV**	0.034	0.036
